# A Review of SiC Sensor Applications in High-Temperature and Radiation Extreme Environments

**DOI:** 10.3390/s24237731

**Published:** 2024-12-03

**Authors:** Quanwei Zhang, Yan Liu, Huafeng Li, Jue Wang, Yuan Wang, Fabin Cheng, Haijun Han, Peng Zhang

**Affiliations:** 1 Institute of Systems Engineering, China Academy of Engineering Physics, Mianyang 621900, China; zhangqw1993@caep.cn (Q.Z.); swcaep@163.com (J.W.); orionwang@foxmail.com (Y.W.); cfb222@163.com (F.C.); hanhaijun@foxmail.com (H.H.); zhangp@caep.cn (P.Z.); 2 The State Key Laboratory for Manufacturing System Engineering, Xi’an Jiaotong University, Xi’an 710049, China

**Keywords:** high temperature, radiation, extreme environment, sensor technology, silicon carbide (SiC), MEMS sensors

## Abstract

Sensors operating in extreme environments are currently a focal point of global research. Extreme environmental conditions, such as overload, vibration, corrosion, high pressure, high temperature, and radiation, can affect the performance of sensors to the point of failure. It is noteworthy that, compared to the resistance to overload and vibration achieved through structural design, the application of sensors under high-temperature and radiation extreme conditions poses a greater challenge. Silicon carbide (SiC) material, due to its excellent physical and chemical properties, such as a large band gap and high atomic critical displacement energy, demonstrates outstanding potential for application in high-temperature and radiation extreme environments. This review presents the current status and research progress of SiC sensors in high-temperature and radiation extreme environments. Finally, given the limited research on the radiation resistance of SiC sensors, it identifies several challenges and research deficiencies in the application of SiC sensors under radiation extreme environments and discusses the future development direction of SiC-based substrate sensors.

## 1. Introduction

With the advent of the information age, sensor technology is assuming an increasingly important role, becoming a strategically developed scientific and technological field in various countries around the world [1,2,3]. This has posed new challenges to the scope of sensor applications and the limits of their performance [4,5,6,7]. Ensuring the reliability and effectiveness of sensors in harsh and severe special environments is a new technical challenge and issue in sensor research [8,9,10,11,12]. Typical special environments involve characteristics such as high temperature, radiation, high vibration, high pressure, and corrosion, with iconic applications in special fields such as aerospace, meteorology, navigation, and weapon equipment. It is worth noting that high-temperature and radiation extreme conditions are difficult to avoid compared to overload and vibration resistance that can be achieved through structural design.

To address the limitations and extremes of sensor applications in extreme environments, it is essential to optimize and improve key aspects such as the selection of functional materials for sensors, structural design, and manufacturing processes. Compared with sensors used in general environments, the research on sensors for extreme environments sets stricter and even more demanding requirements and challenges, from the core sensitive materials and design schemes to the preparation process. As a third-generation semiconductor material, SiC has the advantages of a larger band gap, higher atomic critical displacement energy, resistance to electromagnetic wave impact, and radiation damage, making SiC-based MEMS (Micro Electro Mechanical System) sensors have great potential for application in high-temperature and radiation special environments. However, current domestic and international research on the radiation resistance of SiC electronic devices mainly focuses on power devices such as SBD (Schottky Barrier Diodes), JBS (Junction Barrier Schottky), and MOSFET (Metal Oxide Semiconductor Field Effect Transistor), with less research on the radiation resistance of SiC sensors. Similarly, related research has proven the application potential of SiC materials in the field of radiation-resistant MEMS sensing.

In addition, the mainstream products of sensors that withstand high-temperature and radiation extreme environments are LVDT (Linear Variable Displacement Transducer) sensors, metal-based strain sensors, piezoelectric ceramic sensors, and fiber-optic sensors, but they still have some shortcomings. For example, LVDT sensors have poor linearity and are easily affected by vibrations and impacts in the installation environment [13]; metal-based strain sensors have lower measurement accuracy and weaker resistance to external electromagnetic and temperature interference [14]; piezoelectric ceramic sensors have low measurement accuracy for low-frequency quasi-static signals and poor high-temperature performance [15,16]; fiber-optic sensors have high manufacturing and installation costs, high requirements for light sources and environments, and the difficulty of decoupling output signals, requiring corresponding signal processing equipment, which increases the complexity of the system [17]. SiC has attracted attention as a material due to its excellent performance, especially in high-temperature and corrosive environments. The high hardness and Young’s modulus of SiC material (approximately 450 GPa), as well as the high critical electric field (over 2 MV/cm), make it exhibit better stability and durability under the influence of vibration and impact. In addition, literature shows that SiC piezoresistive pressure sensors exhibit good linearity, high-frequency response, and high accuracy, and the output voltage signal does not require demodulation. Therefore, SiC sensors do overcome the shortcomings of LVDT sensors, metal-based strain sensors, piezoelectric ceramic sensors, fiber optic sensors, and so on in these aspects.

## 2. High-Temperature Extreme Environments

Temperature is an environmental condition that cannot be avoided in all sensor applications. However, apart from temperature sensors that directly utilize temperature-sensitive mechanisms, all other types of sensors are affected and interfere with temperature. At present, the post-processing technology that compensates for the additional temperature effects within the normal temperature range through sensor signals has been relatively mature. Nevertheless, reducing the temperature impact at the source, such as the working principle of the sensor, material selection, structural design, and processing technology, is more targeted and reliable. In some special application fields, such as aerospace, national defense construction, and energy development, there is a need for sensors to work in high-temperature extreme environments, which has led to an increasing market demand for high-temperature MEMS sensors. However, different literature has a large variation in the description of “high temperature”, ranging from about 200 °C to 1500 °C.

### 2.1. Research on the Application of High-Temperature Extreme Environment Sensors

**Figure 1:**
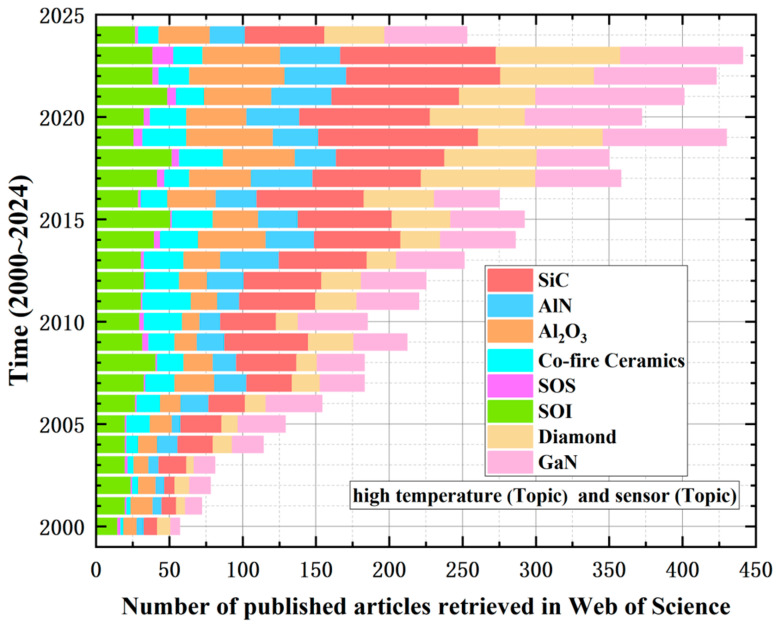
Search results for sensor publications in high-temperature extreme environments.

The characteristics of the materials used for the sensitive chips of sensors largely determine their maximum operating temperature and temperature characteristics. According to the different materials of the chips, high-temperature sensors can be categorized into several types, such as Silicon-On-Insulator (SOI), Silicon-On-Sapphire (SOS), ceramics, diamond, gallium nitride (GaN), and Silicon Carbide (SiC). Utilizing high temperature and sensors as key search terms, a literature retrieval was conducted in the Web of Science (WOS) database. The results, as depicted in Figure 1, reveal that from the year 2000 to 2024, there have been over 6000 published documents. It is evident that the rate and quantity of publications related to SiC have been the highest, underscoring its potential application in high-temperature fields. Subsequently, this paper will discuss the advantages and disadvantages of other MEMS materials for applications in high-temperature extreme environments.

MEMS devices based on silicon material (Si) are the most common, and their material properties, design, process, and testing technology have been thoroughly studied. From a mechanical performance perspective, Si can maintain good elasticity and will not undergo plastic deformation below 500 °C. In terms of electrical performance, because the band gap of Si is only 1.1 eV, the concentration of intrinsic carriers in the Si material will increase exponentially as the temperature rises. Therefore, when the ambient environment exceeds 125 °C, although the mechanical performance is not affected, the reverse-biased P-N junctions used for insulation in Si-based devices or microelectronic circuits will begin to generate leakage current. Thus, when the temperature exceeds 120 °C, the performance of silicon-based sensors will severely deteriorate or even fail. At about 500 °C, silicon-based sensor chips will undergo plastic deformation and current leakage, which cannot meet the measurement requirements of sensors in high-temperature extreme environments.

SOI sensors have high-temperature resistance characteristics and solve the leakage problem. The use of the Silicon-On-Insulator structure can isolate the piezoresistive layer from the silicon substrate layer, avoiding the leakage phenomenon of P-N junctions [18,19]. The key to SOI high-temperature sensors lies in the performance and temperature resistance of the sensitive structure [20,21,22,23,24]. However, unless SOI sensors are combined with a complex high-temperature-resistant packaging scheme, they are difficult to work in environments with higher temperatures (>200 °C) for a long time. SOS sensors, made of Silicon on Sapphire, are less affected by temperature, and their sensitive elements do not experience P-N junction drift [25,26,27]. The operating temperature of SOS sensors generally reaches up to 350 °C, but research has shown that due to the different thermal expansion coefficients between heterogeneous materials, SOS sensors have poor accuracy [28,29,30]. Ceramic materials with high-temperature resistance and insulating properties, such as co-fired ceramics [31,32,33,34], aluminum oxide (Al_2_O_3_) [35,36], and aluminum nitride (AlN) [37], are often made using low-precision manufacturing processes such as ceramic sintering and screen printing for high-temperature sensors. The prepared sensitive chips have issues such as large size, poor accuracy, high leakage rate, low sensitivity, and poor consistency, making it difficult to achieve the engineering and mass production of sensors. Diamond film sensors are also based on the piezoresistive effect of diamonds. Boron-doped diamond films have a high piezoresistive coefficient and strong thermal stability, but the cost of diamond is extremely high, and it has poor compatibility with MEMS processes [38,39,40]. Compared with GaN, which is also widely used in high-temperature MEMS sensors, 4H-SiC has comparable bandgap width, breakdown field strength, and electron mobility. However, 4H-SiC has higher thermal conductivity and elastic modulus, and its oxidation resistance and corrosion resistance are the main advantages compared to GaN. In addition, in the industrial chain, compared with Si, the price of SiC is 30–40 times that of Si, and the price of GaN is 650–1300 times that of Si [41].

### 2.2. Research on the Application of SiC MEMS Sensors in High-Temperature Extreme Environments

SiC (Silicon Carbide) material, as a typical third-generation semiconductor material, is expected to provide a solution to the problem of MEMS device failure at high temperatures. Third-generation semiconductor materials refer to those that have a wider bandgap, strong oxidation resistance, strong corrosion resistance, high physical hardness, high breakdown electric field strength, high thermal conductivity, high electron saturation rate, and high radiation resistance compared to traditional silicon, germanium, and gallium arsenide, which are the first and second-generation semiconductor materials, making them suitable for extreme environmental conditions. A comparison of the performance of SiC with other first- and second-generation semiconductor materials is shown in Table 1 below, from which it can be seen that SiC is several times that of Si and GaAs in terms of band gap width, thermal conductivity, breakdown electric field strength, and hardness.

In the past two decades, the large-scale application of SiC materials in the field of power electronics has catalyzed the prosperity of the emerging semiconductor market, which has promoted the development of manufacturing technology for 4H-SiC and 6H-SiC wafers of various sizes, especially the gradual maturation of homoepitaxy processes [42,43]. On this basis, SiC-based sensors, which are homoepitaxially grown thin-film materials on 4H-SiC or 6H-SiC single crystal substrates for the sensitive layer of sensors, have become a hot research topic in recent years. Based on different sensing mechanisms, the more studied SiC-based sensors can be divided into piezoresistive, capacitive, and fiber-optic types.

#### 2.2.1. Research on Piezoresistive SiC High-Temperature Sensors

Due to the backwardness of processes such as bulk micromachining, the high-temperature piezoresistive sensors made of SiC in the early days mostly used Si, SOI, or sapphire as substrates, with 3C-SiC thin films epitaxially grown on the upper layer as the piezoresistive element layer or isolation layer. Sensors using the piezoresistive structure have a simple structural form and circuit design, are easy to integrate, and are convenient to use, making them the main research direction for early 3C-SiC high-temperature sensors [44,45,46,47,48]. However, the lattice and thermal stress mismatch between 3C-SiC and the Si substrate materials leads to a high density of defects in the 3C-SiC thin film, especially near the SiC/Si interface. Therefore, the hetero-junction formed at the SiC/Si interface suffers from an unacceptable high leakage current at high temperatures; this disadvantage has hindered the application of 3C-SiC piezoresistive sensors at high temperatures [49,50].

**Figure 2:**
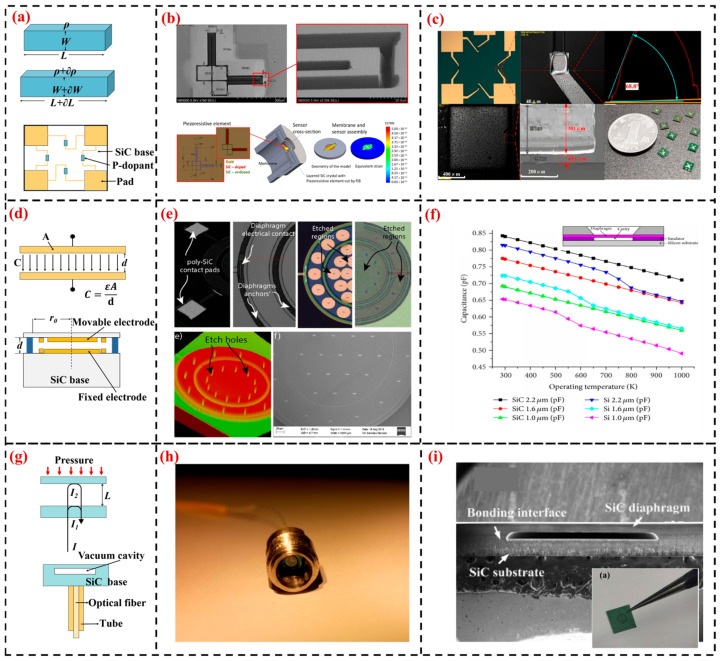
Research on the Application of SiC MEMS Sensors in High-Temperature Extreme Environments: (**a**) Schematic diagram of the principle of piezoresistive sensors; (**b**) High-temperature 6H-SiC piezoresistive sensor from Warsaw University of Technology [54]; (**c**) 4H-SiC piezoresistive sensor from North University of China [56,57]; (**d**) Schematic diagram of the principle of capacitive sensors; (**e**) 3C-SiC capacitive sensor from the University of California, Berkeley [65]; (**f**) 3C-SiC MEMS capacitive pressure sensor [66]; (**g**) Schematic diagram of the principle of fiber optic sensors; (**h**) SiC fiber optic sensor from North University of China [70]; (**i**) All-SiC structure EFPI pressure sensor by Jiang et al. [73].

With the commercialization of SiC materials (4H-SiC and 6H-SiC single crystals) and the increasing maturity of SiC MEMS processes such as epitaxial growth, doping, etching, and metal-semiconductor contacts, high-temperature sensors made entirely of SiC have gradually become the mainstream research direction for piezoresistive sensors, as they use homoepitaxial 4H-SiC and 6H-SiC as chip materials, as shown in Figure 2a, greatly reducing defects caused by heteroepitaxy.

The NASA Glenn Research Center and Kulite Corporation in the United States started their research on all-SiC high-temperature sensors earlier and are relatively advanced in technology. In 2014, Robert S. Okojie and others at the NASA Glenn Research Center [51] achieved a 4H-SiC piezoresistive pressure sensor with an offset voltage as low as 0.125 mV, which can work for 500 h under thermal cycling from 25 to 500 °C. In 2015, Robert S. Okojie’s team [52] reported a 4H-SiC piezoresistive pressure sensor working at 800 °C and found that as the temperature rises from 400 to 800 °C, the full-scale output voltage of the sensor gradually increases to a level similar to that at room temperature.

Researchers at the Swiss Federal Institute of Technology in Lausanne, including Akiyama T, fabricated a 4H-SiC sensor through mechanical grinding [53]. The sensor is capable of operating for 165 h at 600 °C with a sensitivity of 268 μV/(V·bar^−1^). At the Warsaw University of Technology, Wejrzanowski T prepared SiC piezoresistive thin films with different doping elements and concentrations and then utilized Focused Ion Beam (FIB) technology to cut SiC piezoresistive structures. A 316 stainless steel membrane within the package was used to induce the mechanical strain required for the piezoresistive effect, as depicted in Figure 2b. The sensor has a measurement range from 0 to 5 bar, a nonlinearity of 0.48% FS, and the highest test temperature is 300 °C [54].

He Hongtao and others from the 13th Research Institute of China Electronics Technology Group Corporation developed a prototype of a 4H-SiC pressure sensor [55]. The sensor has a measurement range of 700 kPa, a nonlinearity of 1.054%, a sensitivity of 0.00503 mV/(kPa·V^−1^), and the highest operating temperature is 550 °C. At the North University of China, Li and colleagues used Inductively Coupled Plasma (ICP) etching to prepare a P-type 4H-SiC pressure sensor [56,57], as shown in Figure 2c. The sensor has a sensitivity of 1.09 mV/(V·bar^−1^) at room temperature, and the maximum temperature coefficient of resistance (TCR) is −0.08%/°C within the temperature range from 25 to 600 °C.

#### 2.2.2. Capacitive Silicon Carbide High-Temperature Sensor Research

Research in the field of capacitive SiC high-temperature sensors is predominantly conducted at Case Western Reserve University in the United States, where a systematic set of research outcomes has been established [58,59,60]. Domestic research entities include North University of China [61], Aerospace Long-March Rocket Technology Co., Ltd. [62], Xiamen University [63], and others. Notably, Noraini Marsi et al. from Malaysia [64] have also published research findings in this area. It has been observed that the cavity radius is a primary parameter affecting the sensitivity of the device, with larger radii correlating to higher sensitivity.

At Case Western Reserve University, Du J and colleagues utilized phosphosilicate glass (PSG) as a bonding layer material to achieve indirect bonding between polycrystalline SiC thin films and silicon substrates, fabricating a polycrystalline SiC capacitive pressure sensor [58]. The sensor exhibits good linearity within a pressure range of (21.1~26.3) PSIA (Pounds Per Square Inch Absolute), with a sensitivity of 0.62 pF/PSIA at 400 °C and 0.53 pF/PSIA at 500 °C. At the University of California, Berkeley, Beker L and team developed a differential capacitive SiC pressure sensor [65], as depicted in Figure 2e. The concentric ring and diaphragm structure help to eliminate common-mode noise, with a 0.2 μm thick polycrystalline SiC thin film serving as the pressure-sensitive layer. The sensor has a pressure range of (0.5~1.4) MPa, with sensitivities of 1.03 fF/kPa at 20 °C and 0.92 fF/kPa at 180 °C.

Cao Zhengwei and others from Aerospace Long-March Rocket Technology Co., Ltd. developed a 4H-SiC capacitive high-temperature micropressure sensor. The sensor has a range of (0~3) kPa, a sensitivity of 18.7 fF/kPa, nonlinearity of 12.60%, hysteresis of 0.47%, and can operate up to a maximum temperature of 600 °C with an accuracy of 3% across the entire temperature range [62]. Marsi and others discuss the mechanical and electrical effects of 3C-SiC and Si thin films as MEMS capacitive pressure sensors at extreme temperatures of 1000 K [66]. As shown in Figure 2f, the design of thin-film MEMS capacitive pressure sensors using 3C-SiC and Si is compared, with the 3C-SiC thin film exhibiting significantly superior mechanical performance in terms of withstanding higher applied pressures and temperatures compared to Si thin films.

#### 2.2.3. Research on Fiber-Optic Silicon Carbide High-Temperature Sensors

Research on high-temperature SiC fiber optic sensors has been conducted by international scientific teams since the 1970s. Despite the extensive research period, there are still no companies capable of mass-producing commercialized fiber optic Fabry-Pérot high-temperature pressure sensors. Due to the cross-influence of temperature and pressure, the packaging of sensors, and the reliability of the testing systems, most of the well-researched fiber optic Fabry-Pérot high-temperature sensors can only meet some of the testing requirements and cannot fulfill comprehensive testing needs [67].

Pulliam and colleagues from Luna Inc. in the United States bonded SiC thin films to SiC substrates to form Fabry-Pérot cavities and connected sapphire optical fibers to the cavities, extracting the cavity length variation with pressure changes using fiber optic signal demodulation [68]. Subsequently, the University of Florida also developed a fiber optic temperature sensor based on SiC-sensitive structure, and research indicates that the sensor can still operate at temperatures up to 1600 °C. Oxsensis, a company in the UK, has also been working on the development of fiber optic Fabry-Pérot high-temperature pressure sensors and has achieved product commercialization. They are capable of withstanding high temperatures up to 1000 °C for short periods [69] and can operate continuously at 750 °C.

Liang Ting and others from the North University of China used hydrophilic direct bonding technology to achieve sealed bonding between thinned SiC diaphragms and SiC wafers with etched cavities, forming a Fabry-Pérot cavity structure [70], as shown in Figure 2h. The sensor exhibits a repeatability error of 1.42% at room temperature in the (0~800) kPa range. The experiment results show that the sensitivity of the sensor remains at a value of 1.84 nm/kPa at different temperatures (23~400) °C, and the cavity length changes approximately linearly with the temperature with a coefficient of 0.617 nm/°C [71]. Sheng Tianyu and colleagues from Beihang University developed a Fabry-Pérot cavity structure for SiC fiber optic high-temperature pressure sensors [72], capable of measuring pressures in the (0~4) MPa range at 600 °C, with a high-temperature sensitivity of 104.42 nm/MPa and a temperature sensitivity of 0.055 nm/°C. Jiang developed a full SiC-structure thin film based on an EFPI pressure sensor, using nickel diffusion bonding technology to form the sensor element [73], as illustrated in Figure 2i. The sensor shows good linearity in the 0.1–0.9 MPa range, with a resolution of 0.27% F.S. at room temperature, and is expected to operate at temperatures above 1000 °C.

In summary, pressure sensors with different substrate materials and structural types have different performance advantages and relative disadvantages. After decades of development, high-temperature SiC sensors have formed a basic technical theory and solution. The current trend in research on SiC MEMS sensors for high-temperature extreme environment applications is to continuously improve based on existing theories, using high-tech to innovate sensor types and enhance the overall performance of sensors.

## 3. Radiation Extreme Environments

With the increasing application of MEMS devices in fields such as weaponry, nuclear power installations, and space missions, the impact of radiation effects on MEMS devices has garnered growing attention. The focus is primarily on devices made from polycrystalline silicon, monocrystalline silicon, and silicon dioxide, with characteristic dimensions at the nano and micro scales. Radiation (high-energy photons, particles) penetrates the core material layers, transferring energy and causing damage, leading to the degradation of MEMS sensor performance. Nuclear radiation can alter the mechanical and electrical properties of metals and semiconductors. MEMS sensors are used in both ultra-high temperature and strong radiation extreme environments; under extremely high radiation intensities, neutron radiation can even change the molecular structure of sensitive element materials. For instance, with the rapid development of global aerospace technology, especially in the field of outer space, MEMS sensors typically operate in harsh space environments where these devices and systems are susceptible to damage from cosmic rays and various high-energy particle radiation. In weaponry, many different types of MEMS sensors are also used, particularly the force-sensitive sensing units of MEMS mechanical sensors. Radiation can cause structural, electrical, and thermal damage to these sensing units, leading to parameter changes, and performance degradation and thus affecting the stability of the devices [74,75,76]. In high-radiation extreme environments, it can even lead to complete device failure [77,78,79].

**Figure 3:**
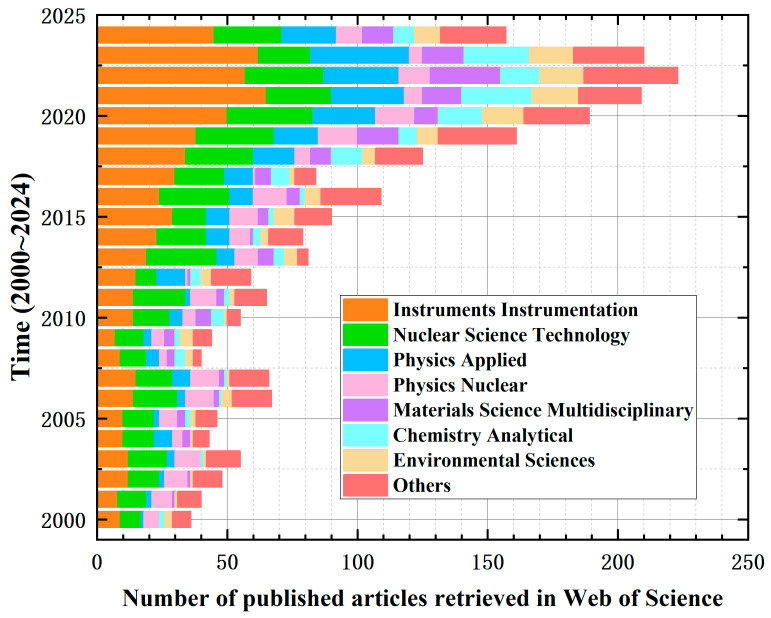
Search results for articles on sensors used in radiation extreme environments.

Using “radiation environment” and “sensors” as key search terms, a literature search was conducted in the WOS database. The results, as depicted in Figure 3, reveal a total of 2322 published documents from 2000 to 2024, of which only 620 articles pertain to sensor devices (instrumentation), accounting for 26.7%. Furthermore, there are only 9 articles specifically addressing the research on SiC sensors in radiation extreme environments, whereas there are 2856 articles on the radiation performance of SiC. This indicates that scholars in the field of sensing technology have overlooked the potential application of SiC sensors in radiation extreme environments, and there is an urgent need for further research. Subsequently, this paper will discuss the existing research on the application of sensors in radiation extreme environments and the application of SiC material radiation performance.

### 3.1. Existing Research on Sensor Applications in Radiation Extreme Environments

H. R. Shea has reviewed the failure of MEMS devices, particularly accelerometers, under various types of radiation and doses in the existing literature [80]. The primary aim is to study the effects of radiation on MEMS devices. MEMS devices operating on electrostatic principles exhibit poor radiation resistance and typically degrade or fail at doses ranging from 30 to 100 krad. In contrast, other sensors with non-electrostatic operating mechanisms show greater radiation resistance, often failing only after several Mrad doses. However, there is limited research on the radiation failure mechanisms of these sensors, making it difficult to determine the failure modes. Consequently, the strength of a sensor’s radiation resistance and its failure mechanisms largely depend on the operating principle of the sensor, with another part depending on the type of radiation source. In devices with weaker radiation resistance, most are MEMS devices based on electrostatic principles unless measures are taken to shield or remove dielectric materials, making the devices insensitive to charges accumulated in the dielectric layer. Tests on accelerometers and RF switches have shown noticeable fluctuations in scale at doses higher than 30 krad. These failures are attributed to charges captured in dielectric thin films. These doses were measured for uncased devices with sensor elements directly exposed to a radiation environment. Similar doses may cause significantly less damage to cased devices. In comparison, piezoresistive MEMS sensors exhibit better radiation resistance, but experiments have observed an increase in the resistance values of piezoresistive devices after radiation, failing at a total dose of 30 Mrad due to the depletion of captured charge carriers. Micro-engines at Sandia National Laboratories in Albuquerque, New Mexico, USA, have been observed to exhibit changes in behavior only at doses exceeding 1 Grad in some cases. These devices contain dielectrics (SiO_2_ and SiN_x_), but the charging at their geometric positions does not directly affect the MEMS devices.

**Figure 4:**
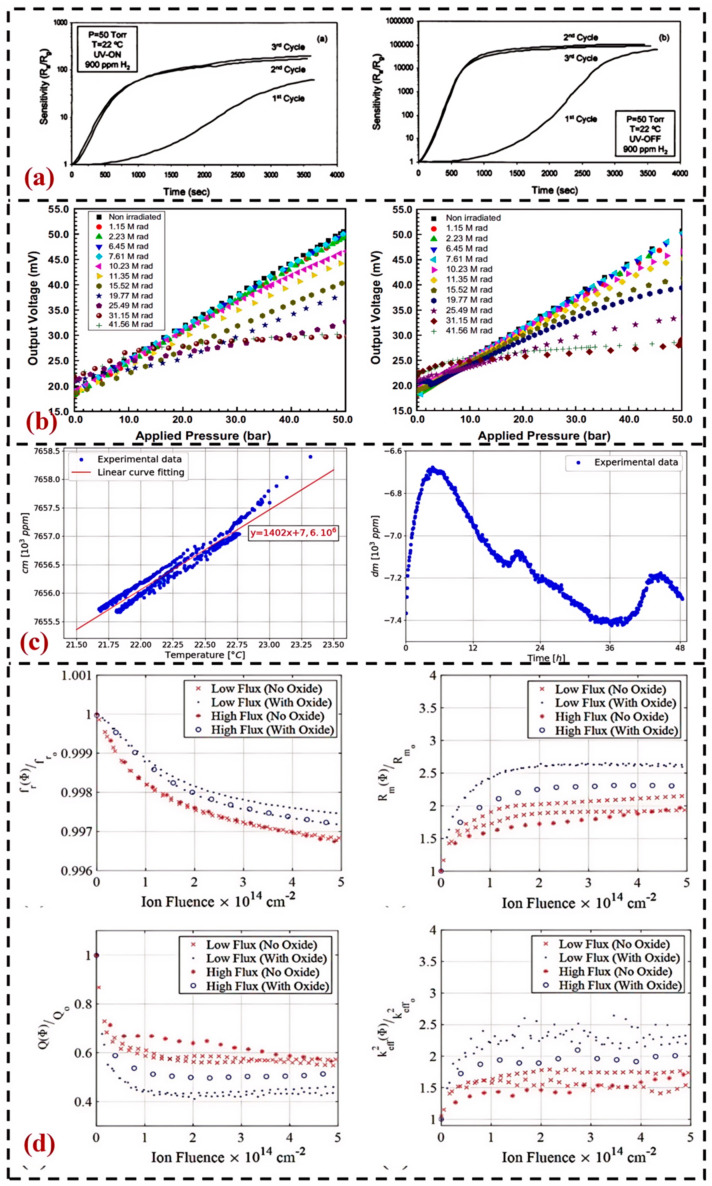
Research on the Application of Current Radiation Extreme Environment Sensors: (**a**) Changes in Sensor H_2_ Sensitivity over Time under Ultraviolet Radiation [82]; (**b**) Trend chart of electrical performance changes of pressure sensor under total dose [84]; (**c**) The effect of gamma radiation on suspended silicon nanogrid bridge used for MEMS transducers [86]; (**d**) The interaction between ion irradiation and piezoelectric conversion MEMS resonators [89].

Damian G. Marinaro et al. studied the effects of proton radiation on MEMS silicon strain gauge piezoresistive elements [81]. The team irradiated two silicon strain gauges with proton radiation. The silicon strain gauges were exposed to a proton fluence of *Φ* = 1 × 10^16^ cm^−2^, and the results showed that the average resistance of the piezoresistors increased from 9.1 kΩ ± 0.5 kΩ to 26.5 kΩ ± 0.5 kΩ. The average resistance change observed after the experiment was approximately 20 kΩ × 10^16^ cm^−2^. Satyajit examined the effect of ultraviolet (UV) radiation on the room-temperature hydrogen (H_2_) sensitivity of gas sensors based on nanocrystalline indium oxide (In_2_O_3_)-doped tin oxide (SnO_2_) thin films [82], as depicted in Figure 4a. The sensor demonstrated a high sensitivity to 900 ppm H_2_, ranging from 65,000 to 110,000 at ambient temperature. Under UV irradiation, the sensitivity to H_2_ was reduced to 200, attributed to the decreased surface coverage of chemisorbed oxygen ions due to UV exposure. E. Bryn Pitt et al. studied the effects of γ radiation on the ADXL325 accelerometer based on a microelectronic mechanical system (Analog Devices, USA) [83]. The radiation-induced performance degradation of the accelerometer within the working range of ±1 g was characterized ex situ and the changes in the input and output relationship of the accelerometer caused by radiation before failure were analyzed. The degradation effects are different on each axis of a single device; as the total dose increases, sensor degradation is manifested as a non-monotonic movement of zero-gravity bias (intercept) and sensitivity (slope); although there are parameter shifts, the input/output relationship between acceleration and accelerometer output remains linear within the acceleration measurement range as the total dose increases.

Vinod Belwanshi et al. studied the degradation of sensors caused by γ radiation by exposing piezoresistive pressure sensors to γ radiation in a Co-60 chamber [84]. The pressure sensors were kept in the Co-60 chamber for a specific duration at a dose rate of 1.05 krad/min. Measurements of γ radiation exposure were carried out up to a total dose of 40 Mrad, at which the pressure sensor permanently failed. As shown in Figure 4b, provides the average magnitude and standard deviation of the four piezoresistors with increasing total radiation dose, indicating that the piezoresistors are not sensitive to γ-ray irradiation and show no significant change. After γ radiation exposure, the pressure sensor’s response to increasing and decreasing pressure is different. Compared to what was observed at lower doses, the response of loading and unloading at a total dose of 20 Mrad or higher deviates more from before radiation. The pressure sensor shows insignificant performance changes at a total dose of up to 10 Mrad (γ rays). However, beyond this dose, the sensor exhibits various parameter degradations, such as reduced sensitivity, increased hysteresis, increased nonlinearity of response, and increased edge of offset voltage. The sensitivity of the sensor significantly decreases after exposure to 10 Mrad, and it further decreases by about 75% after exposure to more than 30 Mrad.

Scholars such as Sun Jing from the Chinese Academy of Sciences conducted research on the radiation response characteristics of irradiated sensors based on the SOI structure [85]. The experiment studied the radiation response characteristics of P-type metal-oxide-semiconductor field-effect transistors with buried oxygen layers on the insulator, including the impact of radiation bias on radiation response sensitivity, differences in sensitivity response in different radiation dose rate environments, and the annealing characteristics after irradiation and their impact on radiation response sensitivity. P. Janioud et al. studied the effects of γ radiation on suspended silicon nano-mesh bridges for MEMS transducers [86], as shown in Figure 4c. The study evaluated the radiation resistance of MEMS sensors based on piezoresistive transducers under γ radiation with a dose rate of 600 Gy/h (total dose of 100 kGy). The results showed that the resistivity sensitivity of a single nanotube was 16.5 ppm/kGy. The paper indicated that the radiation effect on the resonant frequency could be eliminated by differential measurement at both ends of the nano-mesh.

Liu Chaoming et al. from Heilongjiang University conducted research on the electronic radiation effects of capacitive Si MEMS accelerometers [87]. Through 1 MeV electron radiation experiments, the study investigated the regularity of the effects of high-energy electron radiation on the electrical performance of capacitive MEMS accelerometers and related degradation mechanisms. The results showed that the electron radiation fluence had a weak effect on the capacitance of the capacitive MEMS accelerometer, and the impedance was negatively correlated with the radiation fluence; at low radiation fluences, the ASIC circuit of the capacitive MEMS accelerometer worked normally, but significant performance degradation occurred at radiation fluences above 3.0 × 10^13^ cm^−2^; the entire capacitive MEMS accelerometer failed at an electron radiation fluence of 5.0 × 10^12^ cm^−2^.

Scholars, including Fan Linjie from the Chinese Academy of Sciences, conducted experimental research on the total dose effect of ^60^Co γ-ray irradiation on flexible substrate graphene Hall sensors [88]. The electrical performance of the flexible substrate graphene Hall sensor was measured and analyzed before and after γ-ray irradiation up to 1 Mrad. After γ-ray irradiation, there was a slight decrease in the Hall voltage, linearity, offset voltage, and current-related sensitivity of the sensor, with the current-related sensitivity dropping by about 8%. The main reason is the introduction of a small number of impurities and defects into the sensor after irradiation, leading to slight changes in the uniformity and symmetry of the device. Lynes investigated the interaction between silicon ion irradiation and the piezoelectric transduction of MEMS resonators [89], designing and fabricating aluminum nitride (AlN-on-Si) and AlN-SiO_2_-Si bulk acoustic wave (BAW) resonators. These devices were irradiated with 2 MeV Si ions at various fluxes, with a total fluence up to 5 × 10^14^ cm^−2^, and scattering parameters were measured. Specific damage coefficients were derived to describe the radiation effects on the resonant frequency (fr), quality factor (Q), motional resistance (Rm), and electro-mechanical coupling factor (ff_2_). Additionally, damage coefficients for the elastic modulus (E) and piezoelectric coefficient (d_31_) were obtained, conforming to an exponential decay model, as illustrated in Figure 4d.

### 3.2. Existing Research on the Radiation Performance of SiC Materials

The semiconductor properties of materials, particularly their radiation resistance, largely determine the overall radiation tolerance of sensors. As a third-generation semiconductor material, silicon carbide (SiC) boasts excellent mechanical and chemical properties, a wide band gap, and a high atomic displacement energy threshold, demonstrating strong resistance to electromagnetic pulse effects and a high capacity for withstanding radiation damage. This makes it highly promising for use in radiation extreme environments. Generally, the average energy required to generate an electron-hole pair in semiconductor materials is about 3 to 5 times their band gap width [90]. The band gap of SiC is 2 to 3 times wider than that of silicon, indicating a stronger radiation resistance. Moreover, the critical displacement energy of semiconductor materials also characterizes their resistance to radiation-induced displacement damage. The critical displacement energy of SiC is about twice that of Si, further confirming the potential of SiC for radiation resistance [91]. However, current research on the radiation effects of SiC semiconductor performance mainly focuses on SBDs [92], JBS [93], and MOSFET [94] devices. Although the working principles of SiC power devices and their sensors differ, the underlying mechanisms both leverage the semiconductor properties of SiC, such as lattice, carriers, P-N junctions, etc., and these studies have demonstrated the potential application of SiC materials in the field of radiation-resistant MEMS sensing [95].

**Figure 5:**
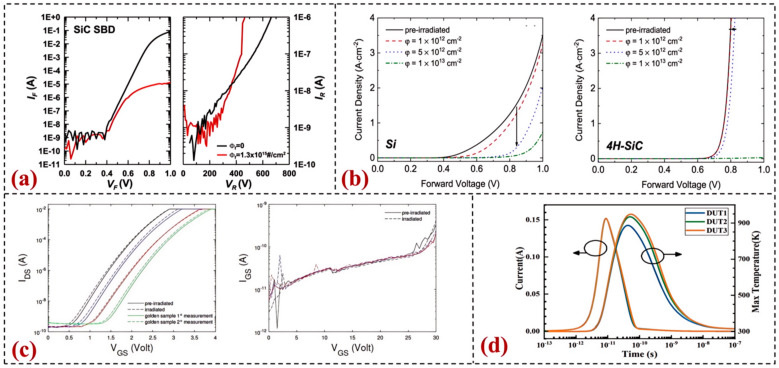
Research on the Radiation Performance of SiC Materials: (**a**) Forward and reverse I-V curves of SiC devices before and after neutron irradiation [93]; (**b**) Forward IV characteristics of Si and 4H-SiC SBDs after 1 MeV proton irradiation [98]; (**c**) Output characteristic curves of SiC MOSFETs before and after neutron irradiation [99]; (**d**) TCAD simulation results of the transient response of the DUT under a 250 V reverse bias to heavy ion impact [100].

Yugo Kobayashi et al. investigated the γ-ray irradiation effects on motor drive circuits composed of SiC MOSFETs under different PWM frequencies [94]. When the PWM frequency was 10 kHz and the irradiation amount exceeded 1.1 MGy, the drive current and voltage wave still remained normal, and the motor continued to rotate at this total dose. SHIH conducted neutron and 60Co-γ irradiation experiments based on 4H-SiC SBDs [92]. The experimental results showed that the electrical performance of SiC devices did not exhibit significant degradation under low-flux fast neutron irradiation. A total dose of 300 krad γ irradiation was used, and its impact on the electrical performance of SiC devices was not significant. Under neutron radiation with a flux of 1.3 × 10^15^ cm^−2^, as shown in Figure 5a, there was a clear change in the I-V curve after irradiation, indicating that a high density of defects was formed within the device.

Gong Min from Sichuan University studied the total dose effect of γ irradiation on a domestically commercial 1200 V SiC power MOSFET device [96]. The output characteristics and transfer characteristics were tested under different total dose irradiations and different environmental temperatures, and the influence of γ irradiation and environmental temperature on the threshold voltage and drain saturation current of SiC power devices was explored. The experimental results showed that the output characteristics and operating state were significantly affected by the irradiation dose, but the device performance partially recovered after room temperature annealing.

Wang from Harbin Institute of Technology conducted a space electron irradiation effect test and analysis on 4H-SiC junction barrier Schottky diodes (JBS) using 1 MeV electron irradiation [93]. The CASINO software was used to simulate the penetration of 1 MeV electrons through SiC samples. The experimental results showed that as the irradiation electron fluence increased, the forward characteristics of the SiC JBS gradually degraded; with the series resistance gradually increasing from 49.8 mΩ to 81.2 mΩ, the free carrier concentration decreased with the increase in irradiation fluence, with a carrier removal rate of about 0.37 cm^−1^. Analysis of the SiC epitaxial wafer material before and after irradiation indicated that electron irradiation causes bulk defects in 4H-SiC, such as carbon interstitial atoms, carbon vacancies, and other complex defects.

Amna used the non-ionizing energy loss mechanism and compared the radiation resistance of Si SBD and 4H-SiC SBD by computer-aided design (TCAD) simulation [97], as shown in Figure 5b. After selecting the same design points, the radiation effects of the two devices were simulated through the interaction of the drift layer, doping, and thickness for a fair comparison. Device simulation results showed that under proton irradiation, 4H-SiC has better radiation resistance than Si, and the performance of Si and 4H-SiC diodes began to decline at proton fluences of 5 × 10^11^ cm^−2^ and 7 × 10^12^ cm^−2^, respectively. Fabio Principato conducted accelerated neutron irradiation experiments on the Chipir-ISIS (Didcot, UK) facility [98], as shown in Figure 5c. Neutron tests were conducted on ST’s SiC power MOSFET using different neutron test systems. The experimental results showed no difference between unirradiated devices and devices that survived neutron irradiation up to a neutron fluence of 2 × 10^11^ n/cm^2^.

Guo from Xiangtan University conducted research on the single-event effect, total dose effect, and displacement damage effect of SiC power devices based on SiC JBS and SiC MOSFET [99]. When SiC JBS and SiC MOSFET were subjected to 20–80 MeV proton irradiation and the devices burned out, the process of the device becoming short-circuited was accompanied by the generation of pulse currents with a wavy shape. Total dose irradiation experiments were carried out using a Co source, and after reaching 500 krad, the static parameters such as the threshold voltage and saturated leakage current of the devices showed significant degradation. At a neutron fluence of 1 × 10^14^ n/cm^2^, both the forward conduction current and reverse current of the SiC JBS decreased.

Wu et al. conducted a detailed study on the heavy ion radiation response and degradation of SiC junction barrier Schottky (JBS) diodes with varying P^+^ implant spacing (S) [100], as depicted in Figure 5d. The experimental results indicate that the larger the S, the more rapidly the reverse leakage current increases and the more severe the degradation after irradiation. TCAD simulations suggest that the electric field at the sensitive spots directly influences the degradation rate of devices with different structures. The large transient energy introduced by heavy ion impact can cause a localized increase in device temperature, leading to lattice damage and the introduction of defects. The reverse leakage current of the degraded devices remains the same as before at low voltages, gradually becoming dominated by space-charge-limited conduction (SCLC) as the voltage increases and ultimately exhibiting ballistic transport characteristics at high voltages.

In summary, scholars have conducted extensive research on the radiation damage effects on the performance of SiC semiconductors, mainly focusing on the changes in the output characteristics of SiC-based SBD, JBS, and MOSFET power devices after radiation. Radiation causes a high density of defects in SiC SBDs, reducing the free carrier concentration. In SiC JBS devices, radiation changes the lattice structure and introduces defect energy levels within the band gap, reducing the carrier mobility and concentration in the material. In SiC MOSFETs, radiation generates a large number of electron-hole pairs in the oxide layer. The aforementioned research results demonstrate the potential of SiC semiconductor performance for radiation resistance, which is necessary for the study of radiation-resistant special MEMS sensing technology.

## 4. Conclusions

In the field of high-temperature extreme environments, current domestic and international research on SiC MEMS sensors has reached a relatively mature stage. With the booming development of the third-generation semiconductor industry, the preparation of MEMS sensors resistant to high temperatures and with high-frequency response, primarily based on all-SiC (4H-SiC and 6H-SiC) materials, is gradually becoming a mainstream research direction for high-end equipment. The trend in the application research of SiC MEMS sensors in high-temperature extreme environments is to continuously improve upon the existing theoretical foundations using advanced technologies, achieve innovation in sensor types, and enhance the overall performance of the sensors. However, there is a significant difference in the performance indicators of existing research on high-temperature resistant SiC sensors, especially in terms of the upper limit of temperature applications, which results in insufficient release of SiC high-temperature performance. The following aspects need to be addressed:

(1) Effective methods are needed to study the impact of specific design schemes on key performance indicators such as sensor range, sensitivity, and zero temperature drift and to clarify the mapping law between spectral parameters and sensor output characteristics;

(2) The main means of achieving precise patterning of SiC transducer components to maximize the optimization of chip design is to optimize the etching process with controllable rate and precise effect. The extremely strong mechanical strength and chemical stability of SiC have brought great problems to the processing technology of sensors, seriously restricting the manufacturing efficiency and surface quality of the core structure and requiring optimization research;

(3) SiC materials have laid the foundation for improving the high-temperature characteristics of pressure sensors, but the overall performance of the device is often significantly affected by the packaging form. Further research is needed on sensor packaging forms to alleviate thermal stress in sensor structures and improve overall temperature resistance and frequency response.

In the field of radiation extreme environments, existing literature on the radiation effects on MEMS sensors has mainly focused on commercial Si and SiO_2_ devices, which inherently have poor resistance to radiation extreme environments. Research on the radiation resistance of SiC electronic devices has concentrated primarily on power devices such as SBDs, JBSs, and MOSFETs, with less research on SiC sensors. However, related studies have demonstrated the potential application of SiC materials in the field of radiation-resistant MEMS sensing. Scholars have established a certain research foundation regarding the damage to SiC semiconductor performance under radiation, which can provide theoretical guidance for related studies. Nevertheless, there are still some deficiencies:

(1) A mathematical description of the micro-scale damage effects of radiation on SiC-sensitive resistors has not yet been established, and it is not possible to construct a unified framework model that comprehensively considers radiation damage and the piezoresistive effects of SiC;

(2) The correlation mechanism between the microstructural damage of SiC-sensitive resistors due to radiation and their macroscopic piezoresistive performance changes is unclear, which does not support the quantification assessment demands for the evolution of SiC device piezoresistive performance under radiation extreme environments;

(3) The mapping rules between SiC process parameters and key parameters of radiation damage are not yet clear, which hinders targeted research on methods to enhance the radiation resistance characteristics of SiC-sensitive resistors.

## Figures and Tables

**Table 1:** Comparison of performance between SiC and two other first- and second-generation semiconductor materials [41].

Unit	Si	GaAS	3C-SiC	4H-SiC	6H-SiC
Bandgap width eV	1.12	1.43	2.4	3.2	3.0
Density g/cm^3^	2.3	5.3	3.2	3.2	3.2
Thermal conductivity W/(cm·K)	1.5	0.5	5.0	5.0	5.0
Maximum temperature resistance °C	600	760	1250	1580	1580
Breakdown electric field mV/cm	0.2	0.3	2.0	2.2	2.5
Young’s modulus GPa	190	75	448	448	448
Hardness kg/mm^2^	1000	600	3980	2130	-

## Data Availability

No new data were created.
